# Airway microecology in rifampicin-resistant and rifampicin-sensitive pulmonary tuberculosis patients

**DOI:** 10.1186/s12866-022-02705-9

**Published:** 2022-11-29

**Authors:** Xingshan Cai, Yang Luo, Yuanliang Zhang, Yuan Lin, Bitong Wu, Zhizhong Cao, Zuqiong Hu, Xingyi Wu, Shouyong Tan

**Affiliations:** 1 Department of Medical Laboratory, Guangzhou Chest Hospital, Guangzhou, 510095 P. R. China; 2 Department of Tuberculosis Internal Medicine, Guangzhou Chest Hospital, Guangzhou, 510095 P. R. China

**Keywords:** Tuberculosis, Microbial flora, 16S rRNA gene sequencing

## Abstract

**Background:**

Pulmonary tuberculosis is a chronic infectious disease of the respiratory system. It is still one of the leading causes of death from a single infectious disease, but it has been stuck in the study of a single pathogen. Recent studies have shown that many diseases are associated with disruption of the native microbiota. In this study we investigated the occurrence of tuberculosis and the correlation between drug resistance and respiratory flora. High-throughput 16 S rRNA gene sequencing was used to characterize the respiratory microbiota composition of 30 tuberculosis (TB) affected patients and compared with 30 healthy (H) controls. According to their Gene Xpert results, 30 pulmonary tuberculosis patients were divided into 12 persons in the drug-sensitive group (DS0) and 18 persons in the drug-resistant group (DR0). The microbial flora of the two were compared with the H group.

**Results:**

The data generated by sequencing showed that *Firmicutes*, *Proteus*, *Bacteroides*, *Actinomyces* and *Fusobacterium* were the five main bacterial phyla detected, and they constituted more than 96% of the microbial community. The relative abundances of *Fusobacterium*, *Haemophilus*, *Porphyromonas*, *Neisseria*, *TM7*, *Spirochetes*, *SR1*, and *Tenericutes* in the TB group was lower than that of the H group, and *Granulicatella* was higher than the H group. The PcoA diagrams of the two groups had obvious clustering differences. The Alpha diversity of the TB group was lower than that of the H group, and the Beta diversity was higher than that of the H group (*P* < 0.05). The relative abundance of *Streptococcus* in the DS0 group was significantly higher than that in the DR0 group (*P* < 0.05).

**Conclusion:**

Pulmonary tuberculosis can cause disorders of the respiratory tract microbial flora, in which the relative abundance of *Streptococcus* was significantly different between rifampicin-sensitive and rifampicin-resistant patients.

## Background

Tuberculosis is an ancient respiratory infectious disease and continues to current days. According to the World Health Organization (WHO) “Global Tuberculosis Report 2021” released in October, 2021: There were nearly 2 billion people latently infected with tuberculosis worldwide. In 2020, there were approximately 9.87 million new cases of tuberculosis in the world. The Report also pointed out that about 1.50 billion death cases were found globally in 2020 with the death rate 15%, higher than 14% in 2019. The estimated number of new cases of tuberculosis in China is 833,000, of which about 65,000 were resistant to the main anti-tuberculosis drug -- rifampicin. Tuberculosis remains the leading cause of death from a single infectious disease [1].

*Mycobacterium tuberculosis* causes tuberculosis and is mostly found in the lungs and respiratory tract. In healthy people, the lungs and airways were not sterile environments. There were five major bacterial phyla in lung and airways of healthy people: *Bacteroides*, *Firmicutes*, *Proteobacteria*, *Actinomycetes* and *Fusobacteria* [2]. The microbial flora could act as a biological barrier to prevent pathogen invasion and participate in human immune regulation, which is mutually beneficial to the human body. Therefore, the composition of the microbial flora can be closely related to the occurrence and development of some diseases.

In the treatment of tuberculosis, antibiotics caused variation in the patient’s respiratory microbial flora. Drug-resistant tuberculosis is a difficult problem in the treatment of tuberculosis, and its mechanism is complex [3]. We tried to take this advantage of the close relationship between antibiotics and microbial flora to find relevant microbial markers for early diagnosis of drug-resistant tuberculosis patients.

The 16 S rRNA, one of the components of ribosome, is a key tool for researches of microbial communities [4, 5]. Ribosomal RNA (rRNA) is the most abundant RNA in cells, accounting for about 80% of the total RNA. There are three types of rRNA in prokaryotes. According to their sedimentary coefficients, rRNAs can be divided into 5S, 16S, and 23S [6]. They are combined with different nucleosome proteins to form the large subunit and small subunit of the nucleosome [7]. The four rRNAs of eukaryotes also use similar methods to form the large and small subunits of the ribosome [6]. 16 S rRNA gene sequences contain hypervariable regions which can identify bacteria for it provides species-specific signature sequences [8]. 16 S rRNA was originally used in bacteria identification, which was found lately in reclassifying new species [9], even genera [10]. Currently, 16 S rRNA sequencing method was widely used in medical microbiology and infectious diseases [11].

To this end, we used 16 S rRNA sequencing to characterize the composition and diversity of sputum microflora in patients with tuberculosis infection, and compared them with those in healthy people. Further, we attempted to identify biomarkers for disease diagnosis by comparing the microbiota differences between healthy individuals and tuberculosis patients as well as between rifampicin-sensitive and rifampicin-resistant patients.

## Methods

### Participants

This study used 30 healthy medical staffs as controls and 30 tuberculosis patients in Guangzhou Chest Hospital were selected, 29 males and 31 females, aged 20–60. In the health (H) group, the medical staffs were non-smokers, who had no history of chronic diseases of respiratory system, no history of respiratory infection in the past 3 weeks, no heart, lung, brain and other diseases, allergic diseases; physical examination and chest X-ray showed no abnormality. The sputum smears of patients in the tuberculosis group (TB) were positive for acid-fast bacilli, which were identified as *Mycobacterium tuberculosis* by nucleic acid test, and no anti-tuberculosis therapy was used at the time of enrolment. Exclusion criteria: (1) patients with severe disease (malignant hypertension, uncontrolled diabetes, myocardial infarction, HIV, etc.), malignant tumor, and pulmonary infection on admission; (2) Oral/intravenous antibiotics were used within 3 weeks before enrolment; (3) Limited understanding ability or inability to cooperate with the examiner; (4) Patients requiring long-term oxygen inhalation (> 12 h/ day). All the subjects had signed the informed consent.

According to the rifampicin resistance results of Gene Xpert MTB/RIF in the patients’ initial sputum, we divided the tuberculosis patients into drug-sensitive (DS) group (12 patients) and drug-resistant (DR) group (18 patients). Sputum samples were collected in H group: 3% saline ultrasound spray was used to extract induced sputum from the respiratory tract. Sputum samples (*n* = 60) from the H group and the TB group were sequenced by 16 S rRNA. After sample preparation, DNA extraction, amplification, database building, up-machine sequencing, data filtering, reads splicing into Tags, Tags clustering into OTU and comparing with the database, species annotation and other statistical analyses were conducted.

### Molecular analysis

#### DNA extraction

Each sputum sample was liquefied using 4% NaOH and buffered in pH 6.8 phosphate buffer then was centrifuged and the supernatant was discarded. The DNA was extracted after the precipitate was taken. The microbial community DNA was extracted using NEB next microbiome DNA enrichment kit (New England Biolabs, Ipswich, MA, US) following instructions of the manufacturer. DNA was quantified with a Qubit Fluorometer by using Qubit® dsDNA BR Assay kit (Invitrogen, USA) and the quality was checked by running an aliquot on 1% agarose gel.

#### Library construction

Variable regions V1–V3 of bacterial 16S rRNA gene was amplified with degenerate PCR primers, 8F (5’-AGAGTTTGATYMTGGCTCAG-3’) and 518R (5’-ATTACCGCGGCTGCTGG-3’). Both forward and reverse primers were tagged with Illumina adapter, pad and linker sequences. PCR enrichment was performed in a 50 µL reaction containing 30ng template, polymerase and PCR master mix. PCR cycling conditions were as follows: 94℃ for 3 min, 30 cycles of 94℃ for 30 s, 50℃ for 45 s, 72℃ for 45 s and final extension for 10 min at 72℃ for 10 min. The PCR products were purified with AmpureXP beads and eluted in Elution buffer. Libraries were qualified by the Agilent 2100 bioanalyzer (Agilent, USA). The validated libraries were used for sequencing on Illumina HiSeq platform (BGI, Shenzhen, China) following the standard pipelines of Illumina, and generating 2 × 300 bp paired-end reads.

#### Sequencing and bioinformatics analysis

Raw reads were filtered to remove adaptors and low-quality and ambiguous bases, and then paired-end reads were added to tags by the Fast Length Adjustment of Short reads program (FLASH, v1.2.11) [12] to get the tags. The tags were clustered into OTUs with a cutoff value of 97% using UPARSE software (v7 0.0.1090) [13] and chimera sequences were compared with the Gold database using UCHIME (v4.2.40) [14] to detect. Then, OTU representative sequences were taxonomically classified using Ribosomal Database Project (RDP) Classifier v.2.2 with a minimum confidence threshold of 0.6, and trained on the Greengenes database v201305 by QIIME v1.8.0. The USEARCH_global [15] was used to compare all Tags back to OTU to get the OTU abundance statistics table of each sample.

Alpha and beta diversity were estimated by MOTHUR (v1.31.2) [16] and QIIME (v1.8.0) at the OTU level, respectively. Sample cluster was conducted by QIIME (v1.8.0) based on UPGMA. Barplot of different classification levels was plotted with R package v3.4.1. GraPhlan map of species composition was created using GraPhlAn. Principal Coordinate Analysis (PCoA) was performed by QIIME (v1.8.0). LEfSe cluster or LDA analysis was conducted by LEfSe. Significant Species or function were determined by R(v3.4.1) based on Wilcox-test or Kruskal-Test.

## Results

### Participant characteristics

After excluding patients and medical staff who did not meet the requirements, 30 PTB patients were enrolled, including 12 RIF drug-sensitive patients and 18 RIF drug-resistant patients. Another 30 health medical staff were included in the study. Table 1 outlines the characteristics of the subjects enrolled in the study: the TB patients were similar in age but different from healthy controls, who were younger than the TB patients (Mann-Whitney test, *P* < 0.05); The gender was similar between the two groups (chi-square test, *P* > 0.05). All the patients were Han Chinese and had no underlying diseases. The healthy group had neither PTB infection nor latent infection.

**Table 1:** Characteristics of the enrolled study participants

Group		Han nationality (%)	Age	Sex (%)		Smear positive (%)	PTB recurrence (%)	PTB with comorbidities (%)	IGRA positive (%)
				Male	Female				
TB patients (*n* = 30)	RIF sensitive (*n* = 12)	100	45 ± 14.18	50	50	100	0	0	100
	RIF resistant (*n* = 18)	100	52 ± 8.91	55.6	44.4	100	61.1	0	100
Health medical staff (*n* = 30)		100	36 ± 10.12	43.3	56.7	0	0	0	0

### The bacterial flora of the respiratory sputum specimens of the healthy medical staff and the tuberculosis patients were diverse, with five main phyla: *Proteus*, *Firmicutes*, *Fusobacterium*, *Actinomycetes* and *Bacteroides*

Among the 10,713,472 raw 16 S rRNA readings obtained, 5,090,698 raw Reads were from the TB group samples, and 5,622,774 raw Reads were from H group samples. Curated Reads (*n* = 3,783,786) from the TB group were obtained by filtering process, which removed 25.67% of original sequence Reads. For H group, 4,138,850 curated Reads were retained and 26.39% original sequence Reads were deleted. After removing the primer sequence, the average read length was about 252 bp. The curated Reads were spliced into Tags through the overlap relationship between the reads, and each sample retained 67,519 tags on average. Finally, 600 OTUs were obtained. The TB group had 168 OTUs, and the H group had 70 OTUs. The two groups had 362 shared OTUs. All OTUs used Greengenes comparison to obtain the five main bacterial phyla: *Proteus*, *Firmicutes*, *Fusobacterium*, *Actinomycetes* and *Bacteroides*. Other bacteria were also detected, such as *TM7*, *Spirochace*, *SR1*, *Cyanobacteria*, and *Tenericutes*, which together accounted for < 4% of the total readings analyzed.

### The TB group had significant difference from the H group in both Alpha and Beta diversity. However, there was no significant difference in Alpha and Beta diversity between DS0 group and DR0 group

**Fig. 1:**
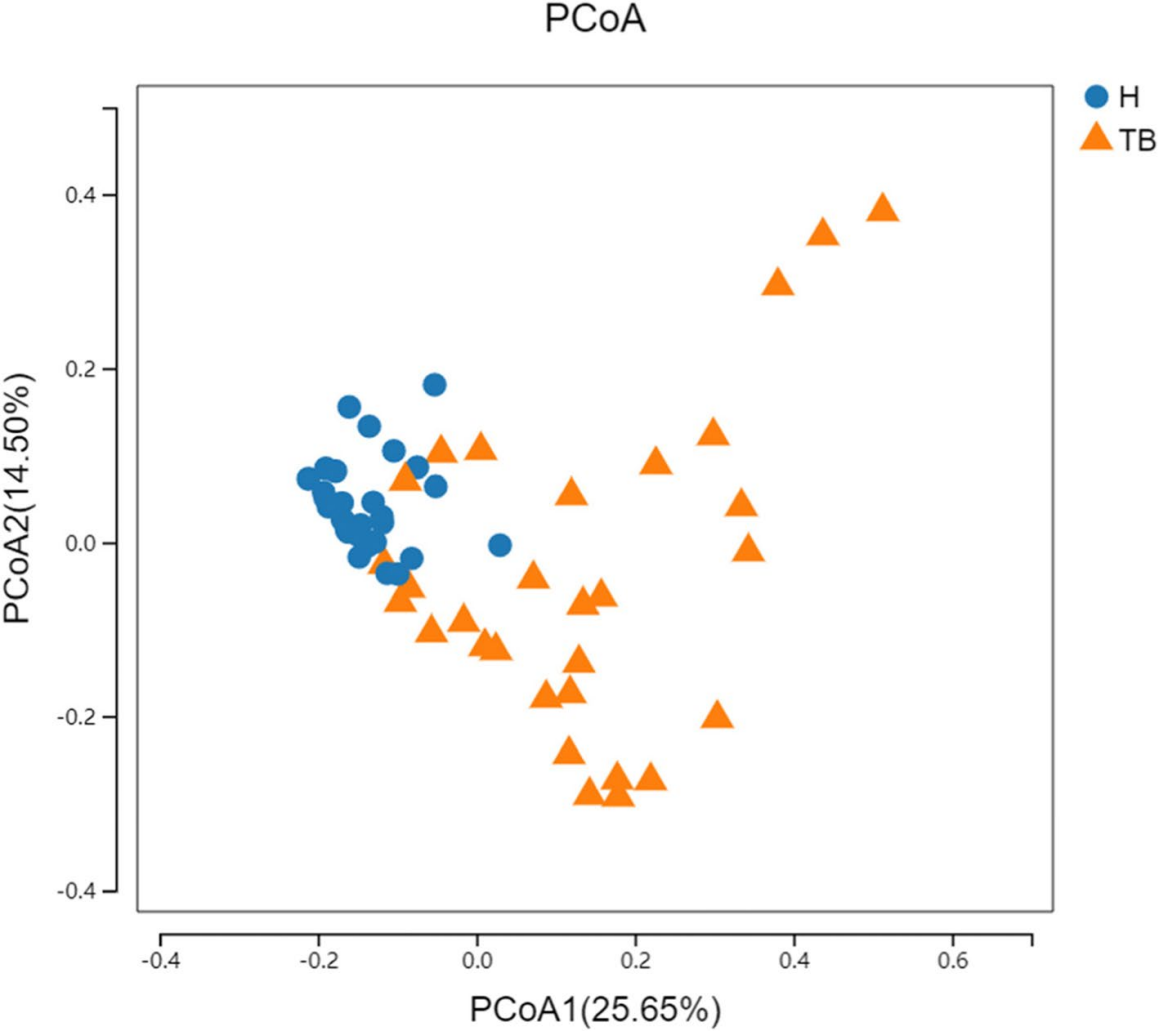
Principal Coordinate Analysis (PCoA) graph based on weighted UniFrac distance. PCoA primary coordinate analysis based on Unweighted UniFrac distance. PCoA1 25.65%, PCoA2 14.55%. (blue: H group, orange: TB group)

**Fig. 2:**
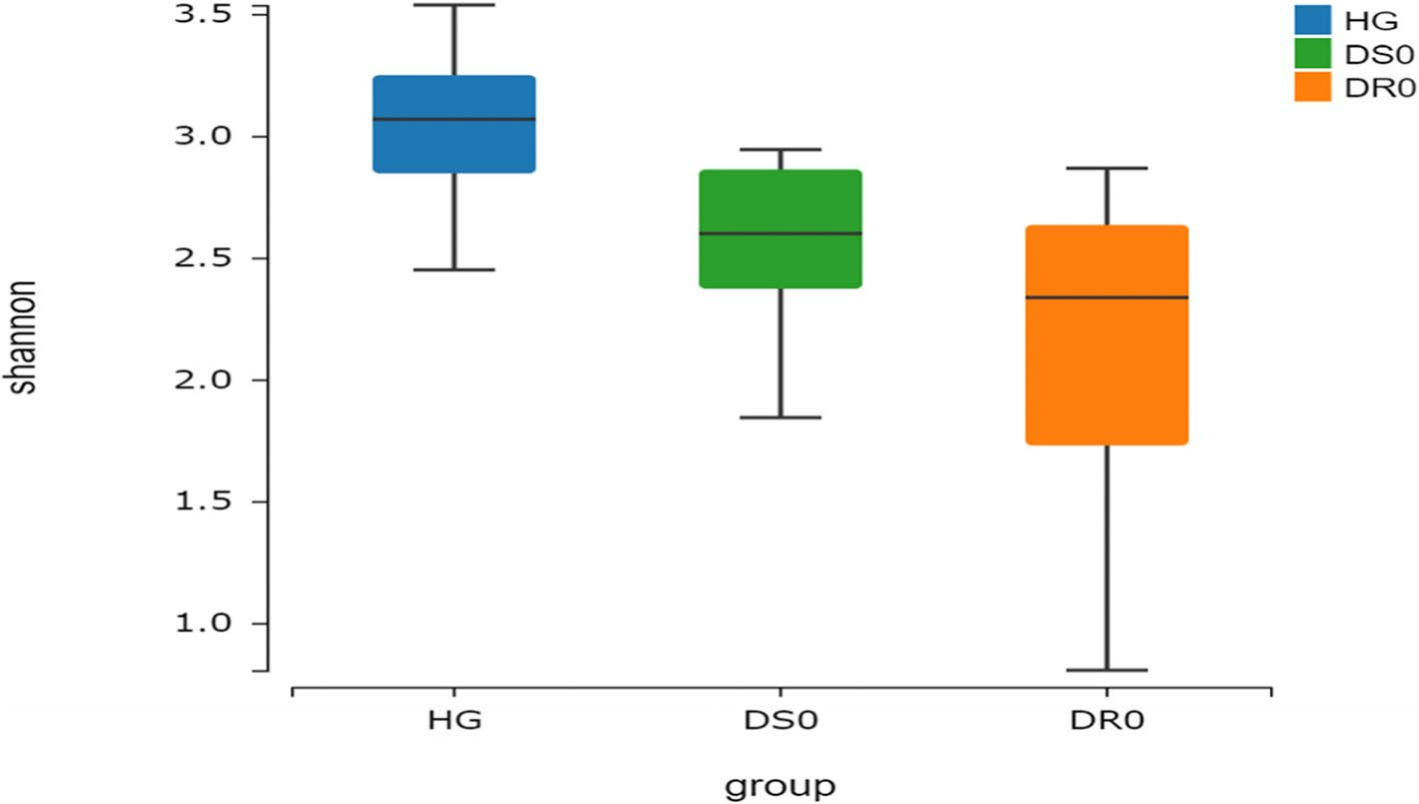
The Alpha diversity of DS0 group, DR0 group and Health control group. Shannon index analysis of bacterial diversity showed that the bacterial flora of DS0 and DR0 groups were lower than those of H group (*P* < 0.001)

**Fig. 3:**
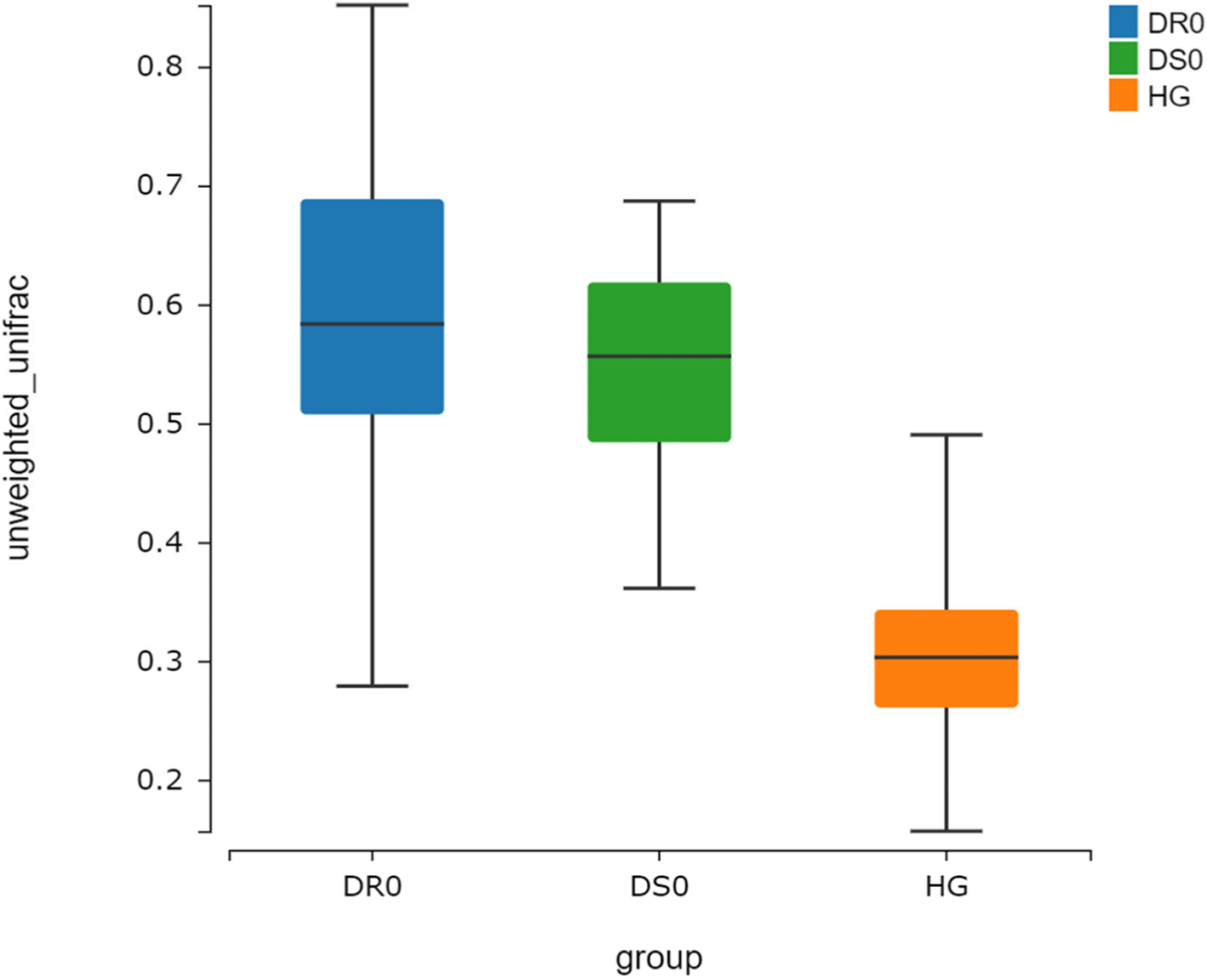
The Beta diversity of DS0 group, DR0 group and Health control group based on unweighted unifrac. The microbial flora of DS0 and DR0 groups were higher than those of H group, respectively (*P* < 0.001)

By phylogenetic-based weighting (Unifrac distance metric), the PcoA principal coordinate analysis between samples in different groups was generated, showing that tuberculosis samples (TB) and samples from health controls (H) have different clusters (Fig. 1). Shannon diversity Kruskal-Wallis Test was used to analyze the differences between the drug-sensitive (DS0) group, drug-resistant (DR0) group and H group. The median values were 2.63, 2.34 and 3.08, respectively, *P* < 0.001. There were statistical differences between DS0, DR0 and H, and the diversity in the samples of TB patients in the above two groups was lower than that of H (Fig. 2). Beta diversity cluster analysis was conducted for samples from the DS0, DR0 and H groups based on the distance between samples, and the median values were 0.56, 0.58 and 0.30, respectively, *P* < 0.001, indicating that there was a statistical difference between DS0, DR0 and H (Fig. 3). The inter-individual diversity of samples from TB patients in the above two groups was higher than that of H. There is no significant difference on both Alpha diversity and Beta diversity between DS0 group and DR0 group (*P* > 0.05).

### There were differences in the relative abundance of species between the tuberculosis and healthy respiratory samples

**Fig. 4:**
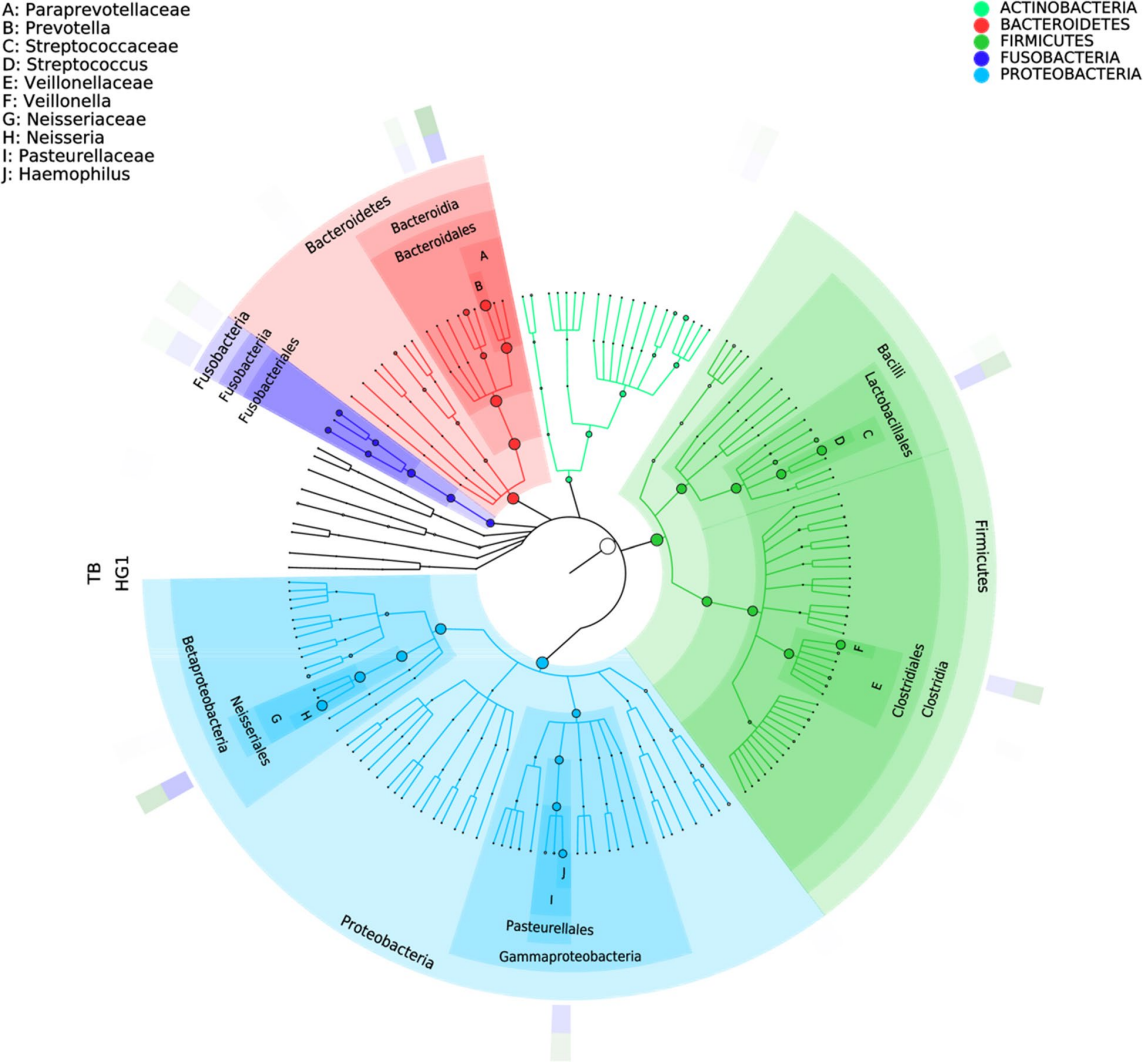
Diagram of dominant species in TB group and H group. The classification level tree displayed by Graphlan described the hierarchical relationship of all the microbial flora from phylum to genus (successively ordered from the inner circle to the outer circle) in the sample community. The node size corresponded to the average relative abundance of the microbial flora, which showed the advantage bacterium group of both TB group and H group. Light green, red, dark green, purple and blue represented *Actinobacteria*, *Bacteroidetes*, *Firmicutes*, *Fusobacteria* and *Proteobacteria* respectively

**Fig. 5:**
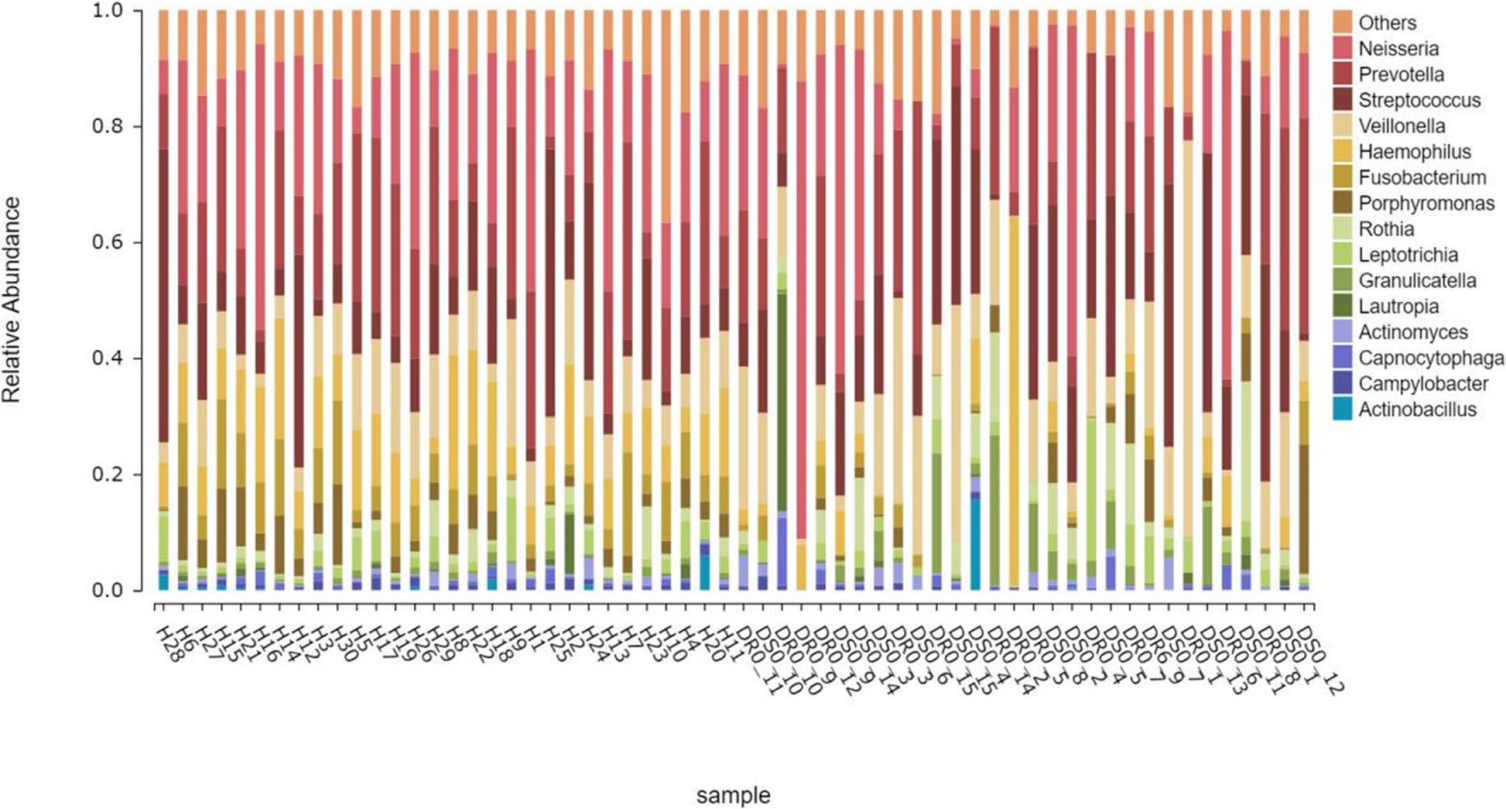
Composition map of bacterial species abundance of all samples in the tuberculosis group (TB) and healthy group (H), mainly 15 genus

Samples (*n* = 60) were collected from both patient group and healthy group, then were sequenced and clustered into 600 OTUs by 16 S rRNA amplicon. Greengenes annotation was used to identify *Actinobacteria*, *Fusobacteria*, *Bacteroidetes*, *Proteobacteria* and *Firmicutes* as five main phyla. The dominant phyla were *Proteobacteria* and *Firmicutes*. The corresponding dominant bacteria genera were *Neisseria*, *Haemophilus*, *Streptococcus* and *Veillonella*. In addition, the main genus of *Fusobacteria* is *Fusobacteriales*, while the main genus of *Bacteroidetes* is *Prevotella* (Fig. 4). On the level of genus, the top 15 species in abundance were *Actinobacillus*, *Campylobacter*, *Capnocytophaga*, *Lautropia*, *Actinomyces*, *Granulicatella*, *Rothia*, *Fusobacterium*, *Leptotrichia*, *Porphyromonas*, *Haemophilus*, *Veillonella*, *Streptococcus*, *Neisseria* and *Prevotella* (Fig. 5).

**Fig. 6:**
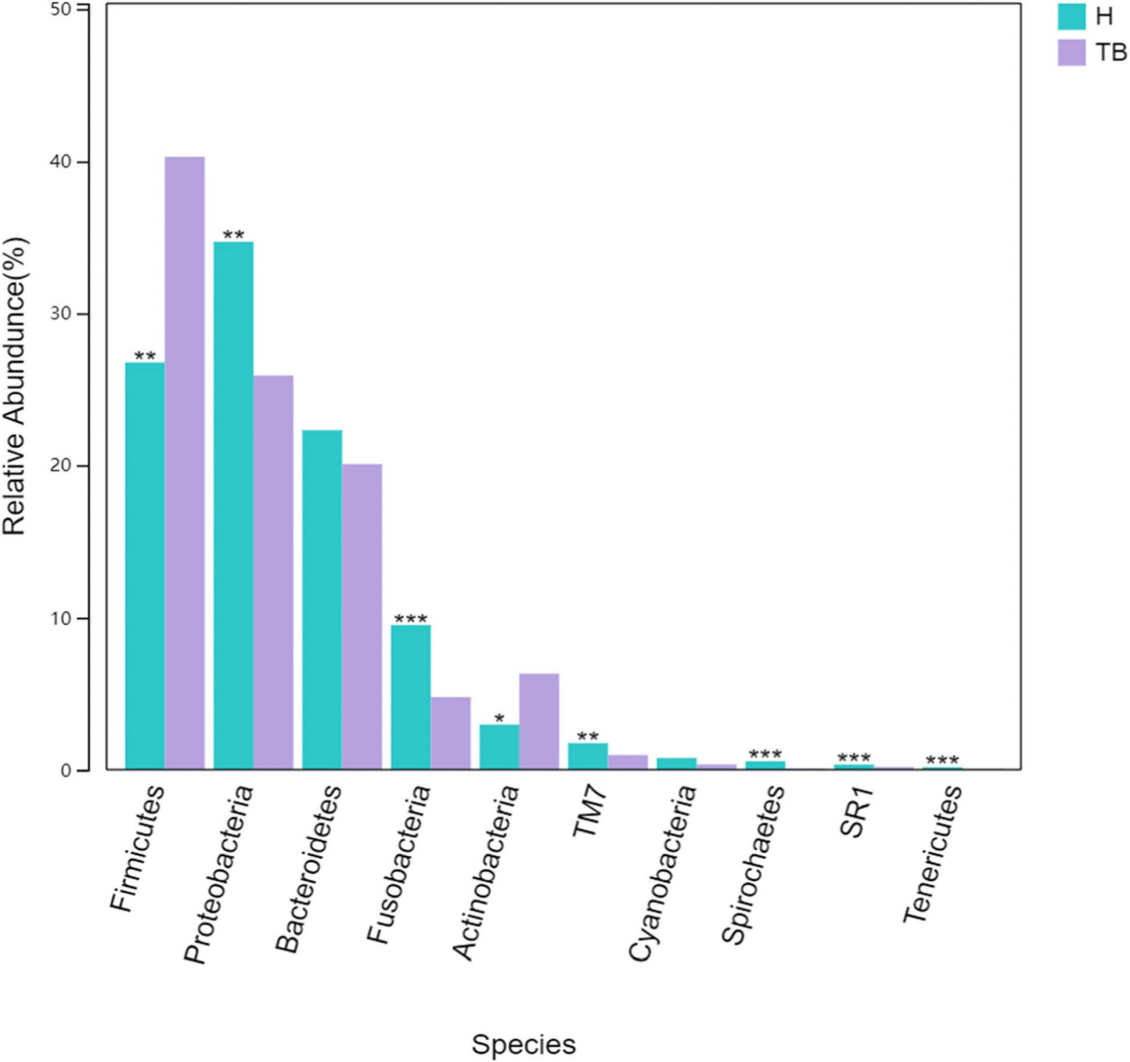
Differences in relative abundance between TB groups and healthy groups in phylum level (top 10). ***, ** and * represent *p*-value < 0.001, < 0.01 and < 0.05 between the two groups, respectively

**Fig. 7:**
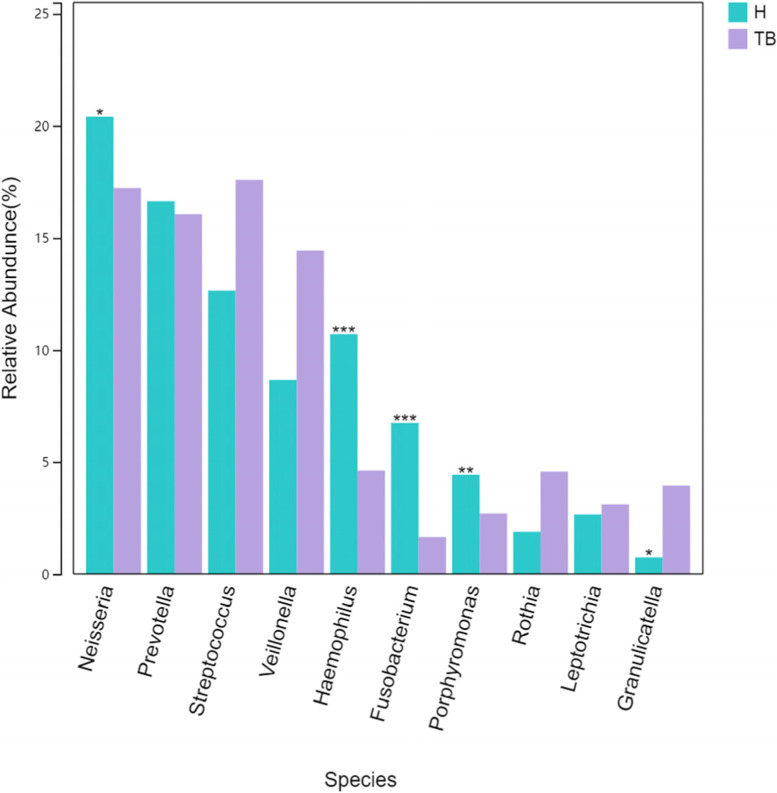
Differences in relative abundance of TB groups and Health group in genus level (top 10). ***, ** and * represent *p*-value < 0.001, < 0.01 and < 0.05 between the two groups, respectively

In phylum level, the relative abundance of *Proteobacteria* (25.89% vs. 36.69%), *Fusobacteria* (4.76% vs. 9.51%), *TM7* (0.96% vs. 1.75%), *Spirochaetes* (0.07% vs. 0.56%), *SR1* (0.18% vs. 0.34%), *Tenericutes* (0.06% vs. 0.16%) in TB group was lower than H group (*P* < 0.05) and the relative abundance of *Firmicutes* (40.30% vs. 26.75%) and *Actinobactria* (6.30% vs. 2.95%) in TB group was higher than H group (*P* < 0.05) (Fig. 6). *Firmicutes* in TB group was mainly increased compared with H group by *Streptococcus *(17.59% vs. 12.64), *Veillonella *(14.43% vs. 8.65%) and *Granulicatella *(3.94% VS 0.74%) (Fig. 7). *Actinobactria* in TB group was mainly increased compared with H group by *Rothia* (4.57% vs. 1.89%), the expression in species level was *Rothia aeria* (0.219% vs. 0.002%). Genus level with relative abundance less than 1.5% in both (TB vs. H) groups included *Turicibacter* (0.0023% vs. 0), *Mycobacterium* (0.03% vs. 0), *Actinobacillus* (0.533% vs. 0.501%) and *Scardovia* (0.032% vs. 0.002%), *Atopobium* (0.34% vs. 0.139%), *Actinomyces* (1.27% vs. 0.689%), *Roseburia* (0.0006% vs. 0), all *P* < 0.05.

The relative abundance of the first 15 bacterial genera in sputum samples of the TB and H groups (Fig. 5), and the differences of key genus were shown in Fig. 7. The relative abundance of *Neisseria*, *Haemophilus*, *Porphyromonas*, *Fusobacterium* and *Granulicatella* were different. The Relative Abundance of the first 4 genera of bacteria in the above two groups were 17.23% and 20.40%, 4.61% and 10.70%, 2.71% and 4.43%,1.65% and 6.74%, respectively. Wilcox rank sum test showed that the *p*-values were 0.04, 0, 0.001, 0, respectively, which indicated that the genera mentioned above in TB group was lower than that in H group. However, *Granulicatella* in TB group is higher than H group: 3.94% and 0.74%, *p*-value was 0.03. At the species level, the relative abundance of *Veillonella_dispar* was 10.82% and 3.75% higher than that of H, and the *p*-value was 0.01. *Neisseria_subflava*, *Haemophilus_parainfluenzae*, *Prevotella_pallens* and *Prevotella_nanceiensis* were lower in TB than in H (*P* < 0.05). It is worth mentioning that *Mycobacterium* accounts for a very small proportion of the entire sputum microbiota, whose relative abundance was only 0.03%.

### There were differences in the distribution of bacteria in the drug-sensitive group (DS0) and drug-resistant group (DR0)

**Fig. 8:**
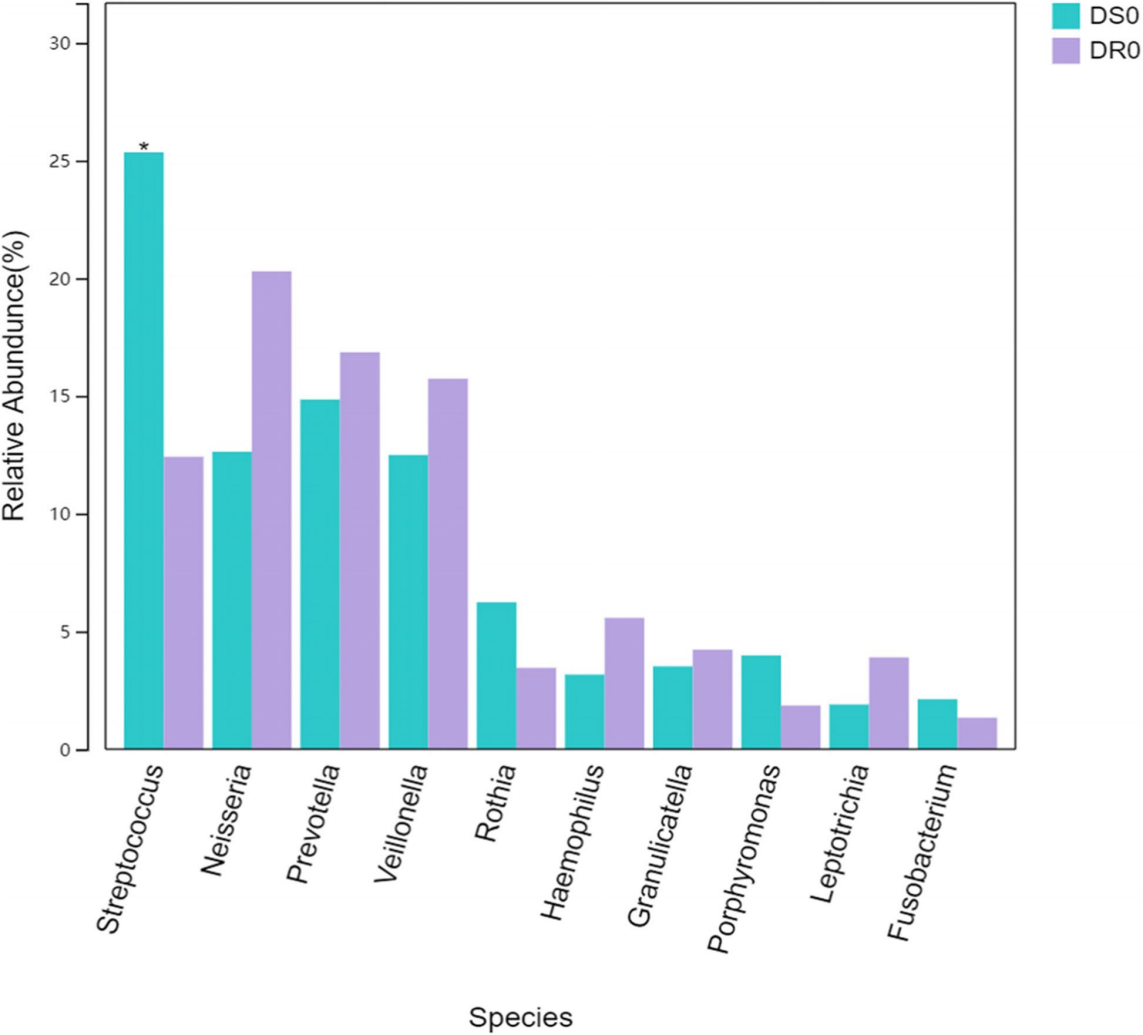
Differences in the relative abundance of bacteria in the respiratory tract samples between the tuberculosis treatment sensitive group (DS0) and the tuberculosis treatment resistant group (DR0), the relative abundance of *Streptococcus* of DS0 was higher than DR0, *P* < 0.05

There were differences in the relative abundance (top 10) of DS0 and DR0 (Fig. 8). The *Streptococcus* relative abundance of DS0 group is higher than that of DR0 group :25.35% and 12.41, mainly reflected in the species level of *Streptococcus_infantis*, *P* < 0.05, showing statistical difference. Moreover, *Lachnoanaerobaculum* (0.03% vs. 0.21%) and *Lautropia* (0.62% vs. 2.23%), which had lower relative abundance, had statistical differences between DS0 and DR0, with *p*-value < 0.05.

## Discussion

After 16 S rRNA sequencing data analysis, the respiratory secretions of 30 tuberculosis patients and 30 healthy medical staffs were clustered into 600 OTU, including five major bacterial phyla: *Proteus*, *Firmicutes*, *Fusobacterium*, *Actinomycetes* and *Bacteroidetes*, and 15 major bacterial genera (Fig. 5), which was similar to most reports [17–19]. There were 168 unique OTU in the TB group and 70 unique OTU in the H group, indicating that there were more exotic species in the TB group, which was similar to that reported by Zelin Cui [19]. PcoA principal coordinate analysis showed that there was clustering difference between TB and H groups, which was shown in both Alpha diversity and Beta diversity. The data showed that the intra - individual diversity of PTB respiratory microbial flora samples decreased, but the inter - individual diversity increased.

The airway microecology is perturbed in many chronic respiratory diseases. In bronchiectasis, the airways became widened and mucociliary clearance fails, resulting in increased adhesion of potential pathogenic microorganisms (PPMs) such as *Pseudomonas* and *Haemophilus* [20]. In chronic obstructive pulmonary disease (COPD), *Haemophilus_influenzae* and *Moraxella* were associated with disease progression and exacerbation [21]. Cystic fibrosis (CF) is associated with infections of *Staphylococcus_aureus* and *Ceratocystis_cepacia* [22]. Our study on the airway microorganism of PTB is slightly different and has no correlation with the PPMs of Proteobacteria mentioned above. The reason is that the diseases mentioned above are chronic infections caused by the increased abundance of colonizing bacteria in the respiratory tract. However, the pathogen of PTB is *Mycobacterium tuberculosis* (MTB), which belongs to foreign bacteria and is caused by MTB breaking through the respiratory barrier and invading the lungs due to the low resistance of the body or the destruction of lung structure or the reduced ability of cilia clearance. Our data showed that no co-infection of other PPMs occurred. In addition, our study showed that the relative abundance of *Firmicutes* and *Actinomycetes* in the airway of PTB increased, while the relative abundance of other phyla in the airway of PTB decreased, which was similar to the report of Jing Wu [23] and P. Krishna [18]. As an opportunistic pathogen, *Rothia* could cause endocarditis, meningitis, and bacteremia in people with low immunity [24]. *Atopobium* was the causative agent of bacterial vaginitis, which could also cause bacteremia in people with low immunity [25, 26]. Some other reports on the respiratory tract of PTB are different from our data, such as Zelin Cui [19] reported that *Stenotrophomonas*, *Cupriavidus*, *Pseudomonas*, *Thermus*, *Sphingomonas* and other foreign bacteria are unique to the airway of PTB; Yuhua Zhou reported [17]: the dominant genus of bacteria in the tuberculosis patient’s lower respiratory tract is *Cuprophyll* rather than *Streptococcus*. In terms of bacterial genera in this study, *Neisseria*, *Haemophilus*, *Porphyromonas* and *Fusobacterium* were lower than those in H group. *Granulicatella* and *Veillonella_dispar* were higher than those of H group. There are 15 Species of unique biomarkers in TB group, which are derived from *Firmicutes*, *Actinobacteria* and *Proteobacteria*. The results are different from the above reports, which may be due to the different sequencing depth and bacteria detected in each report. On the other hand, the environment, region, diet, race and underlying diseases of patients in each report are also different.

The disorder of the microbial flora in the airway of PTB is a second-degree disorder, which is a manifestation of chronic disease, also known as a localized microbial flora disorder, which can slowly recover after rapid removal of MTB pathogens and reduced inflammation. We speculate that after the MTB entering into the lungs, the body played the innate immune function of lungs. Most bacteria were infiltrated by neutrophils and phagocytosed by macrophages, and the immune system of the body could effectively remove them. Adaptive immunity would be activated in a few days, the specific immune amplification, such as T cells, the secretion of inflammatory cytokines, help to remove pathogen. During this process, the strong host immune response may kill or clear the normal bacterial flora in the respiratory tract (such as *Proteobacteria*, *Fusobacteria*, etc.), and at the same time cause inflammation and damage to the lung tissue, resulting in more mucus production and changes in the airway microenvironment such as local hypoxia, temperature rise, pH value and nutrition. It is conducive to the reproduction of Gram-positive bacteria such as *Firmicutes* and *Actinomycetes*. The cell wall of the above bacteria is composed of a thick and dense layer of peptidoglycan and phosphogeicylic acid, and it has a relatively strong resistance to the outside world. The relative abundance of *Mycobacteriaceae* in our study was not high, which was similar to former research. However, as pathogenic bacteria and Gram-positive bacteria, the relative abundance of *Mycobacteriaceae* was obviously correlated with the increase of *Firmicutes* and *Actinomycetes*, which may be potential microbial markers of MTB infection. In addition, the relative abundance of *Granulicatella* increased in TB group. Recently, *Granulicatella* has caused endocarditis, bacteremia, peritonitis, arthritis, etc. [27], and whether *Granulicatella* has caused double infection of respiratory tract is also a concern. For drug-resistant TB patients and drug-sensitive TB patients, Dongzi Lin [28] found that the Alpha diversity and Beta diversity of the two groups were statistically different, and the relative abundance of *Ralstonia*, *Delftia*, and *Neisseria* was increased, which was different from our data. We considered that it was related to the inclusion of different drug resistance groups.Data in Fig. 8 showed that the Relative abundance of *Streptococcus* in DS0 was much higher than that of DR0, mainly *Streptococcus infantis*, which belonged to physiological *Streptococcus virifolia* and was the most powerful colonization bacteria in the oropharynx. Some studies have reported that, this bacterium had an inhibitory effect on the growth of *Streptococcus suppurans*, *Streptococcus pneumoniae* and Gram-negative bacilli [29]. Therefore, when other bacterial genera were not adapted to the external environment and weakened, *Streptococcus* grows rapidly, perhaps because of the increase in the Abundance of this protective bacterium, it indicated a good outcome of the disease. Our studies indicated that *Streptococcus*, principally *Streptococcus infantis*, might be the biomarker of drug-resistant TB diagnosis. DR0 is shown as the disorder of normal microbial flora, with no increase in *Streptococcus* and no increase in other PPMs. Jing Wu [23] found that the frequency and abundance of *Bulleidia* and *Atopobium* were lower in patients with recurrent PTB than in new-found TB patients, while the proportion of *Pseudomonas*/*Mycobacterium* was higher in recurrent TB patients than in new-found TB patients. In our study, the relative abundance of *Lachnoanaerobaculum* and *Lautropia* of 61.1% (11/18) of recrudescent-treated patients in DR0 group was higher than that of the newly treated patients.

Drug-resistant TB patients mostly recurrent patients, had been treated with anti-TB drugs or other antibiotic in the initial treatment period. The imbalance of bacteria in the respiratory tract may be persistent, and drug-resistant MTB was screened out. Any disorder of the normal microbial would destroy the immune response of human body, thus causing the colonization of various pathogens [30, 31]. Therefore, we assumed that the continuous use of antibiotics may increase the selection pressure of respiratory flora and bacterial resistance of drug-resistant TB patients, especially MDR-TB patients, during the follow-up anti tuberculosis treatment for about one year. The difficulty in clearing *Mycobacterium tuberculosis* also aggravated the destruction of the lung structure, causing a vicious circle to the disease. In fact, the effect of our subsequent patients also confirmed this situation: 100% (12/12) of DS0 patients were cured, 22.2% (4/18) of DR0 patients failed treatment, and 16.7% (3/18) of DR0 patients relapsed one year later.

Our study had some limitations, for example, our sample size was too small to represent the overall phenomenon of changes in respiratory microbial flora of PTB. 16 S rRNA sequencing was not long enough to map most sequences to the species level, and next-generation sequencing technologies with longer sequence reads were needed to overcome this problem in future studies. The patient group we studied was older than the control group, and it is possible that the microbial population mutates as humans age.

## Conclusion

The respiratory microecology of tuberculosis patients and health workers had different clustering, diversity of bacteria genus was different, and secondary dysregulation of bacteria community occurred. The increased bacterial load of *Streptococcus* in DS0 group may be one of the indicators of good prognosis of PTB, and the occurrence of bacterial disturbance in DR0 group is closely related to the occurrence and progress of PTB. *Streptococcus_infantis* may be used as a potential diagnostic marker for both. Biological regulation of normal microbial flora may be tuberculosis pathogenesis and drug resistance mechanism was an important issue, pay more attention to ecology, rather than only emphasize single pathogen separation, adjusted by probiotics to respiratory tract microecological, keep it in a healthy state of balance, the host of the potential change is likely to prevent the host - harmful microorganism group interaction comprehensive solution. Therefore, increasing the research on the species and function of respiratory microecology as well as the correlation with diseases can become a new idea for clinical prevention and treatment of PTB.

## Data Availability

All data generated or analysed during this study are included in this published article. Data can be accessed from NCBI Sequence Read Archive (SRA) and the number was PRJNA837186 (www.ncbi.nlm.nih.gov/sra/PRJNA837186).

## Abbreviations

TB: Tuberculosis
RNA: Ribonucleic acid
WHO: World Health Organization
MTB: *Mycobacterium tuberculosis*
RIF: Rifampicin
DNA: Deoxyribonucleic acid
HIV: Human Immunodeficiency Virus
PTB: Pulmonary tuberculosis
PPMs: Potentially pathogenic microorganisms
COPD: Chronic obstructive pulmonary disease
